# Cholera—Latest Discussions in Germany on the Subject

**Published:** 1873-11

**Authors:** 


Article VI. — *Cholera — Latest Discussions in Germany on the Subject.*

The causes of the spread of cholera are of such interest to every one that it seems desirable to briefly notice the latest discussions in Germany on this subject.

The great reputation of Pettenkofer, his acuteness and able statement of his views, have insured his opinions a wide acceptance, and are at the present moment influencing the course of investigation, both in Europe and in India. If, as some suppose, Pettenkofer has been on a wrong track, his influence may unfavorably bias the investigation, and it seems important to exactly define his position and the value of his arguments. He has, in the course of years, changed several of his views, and has been subjected to some sharp criticism in consequence; and lately a very able and thoughtful attack on his ground-water hypothesis, by Dr. F. Sander,* and to some extent by Kuchenmeister, has appeared.†

Pettenkofer has found it necessary to reply to Sander in a long article in his Journal,‡ entitled, "On the present state of the cholera question." In this he has defined his own views, and has discussed with great ability the general question of the spread of cholera.

A brief notice of these three essays will enable us to analyze and prove what Pettenkofer's opinions are at this moment.

Sander's conclusions are these: he does not deny that in the spread of cholera there is, to use the language of Pettenkofer, "a disposition given by time and place," or, as we should say, by conditions of season and locality.

In fact, this is one of the most obvious points in the spread of cholera; in India, the cholera seasons of special places are well known; and the prevalence of cholera in certain spots, and the exemption of others, have long been observed with the greatest interest.

Sander does not again deny that the condition of the soil may play a part in some instances in preventing or favoring the spread, but he considers such a part as non-essential, so to speak, and that the local favoring conditions, whatever they may be, need not be connected with the soil. Beginning his inquiry with a leaning to Pettenkofer's views on this point, he has gradually found it necessary to diverge from him, and has been obliged to attribute a great importance to infection, by means of the intestinal excreta, and to multiplication of the "cholera ferment" in the bodies of men.

Kuchenmeister has come also to pretty much the same conclusion. He considers cholera without men to be inconceivable; epidemics are produced sometimes by direct infection, more frequently by the aid of so-called "helping causes" in the ground.

As means of this spread, we have, he says, in India as in Europe, traffic and intercourse with cholera-sick men and places, and, before everything, proximity to their clothes, foul linen, house

* "Untersuchengen uber die Cholera in ihren Beziehungen zu Boden und Grundwasser." Von Dr. Fr. Sander. Cologne: 1872.

† "Handbuch der Lehre von der Verbreitung der Cholera." Von Dr. F. Kuchenmeister. Erlangen: 1872.

‡ "Zeitschrift fur Biologie." Band viii, p. 492. 1872.

utensils, and privies. The most probable carriers of the infecting matter are considered by Kuchenmeister to be the cholera dejections, and the failure of the so-named disinfection of the dejections in the ground, or in privies, is no proof against the view. That the cholera moves with the wind, Kuchenmeister considers not only unproved but as improbable, and experience is opposed to this view. Starting then from this point Kuchenmeister considers the "helping causes of place and season" to be air-temperature and rain; especially the permeability of the soil for the air-temperature and air-masses in the superficial and deep strata; and after these he considers that the putrefactive changes in the soil are of importance. As to temperature of soil he considers that it is tolerably certain that cholera will not spread if the temperature of the upper strata of the ground which are in relation to men, sinks under 54.5° Fahrenheit. The ground-water plays a part, he thinks, in the cholera spreading, but not that which Pettenkofer ascribes to it; and other conditions, especially the temperature of the soil, and the amount of water in the upper strata, are of much greater importance than the ground-water. With regard to the spread through drinking-water, this has been as often affirmed as denied in Europe as in India. "The worth of this opinion," says Kuchenmeister," is daily lessened among us."

Such, then, are the opinions of these two writers, which differ only from those received commonly in India and in England in the doubt expressed on the spread by drinking-water; a doubt also shared by Pettenkofer.

As already said, Pettenkofer replies especially to Sander in a long article. He refers to his own changes of opinion, and believes such changes to be inevitable with every one. He rates our knowledge of cholera low, but wishes to define his present views as carefully as he can.

He begins with a reference to the old terms of miasma and contagion, which he uses in the sense that "Contagion" is a specific infection-matter arising within the body of the person, and "Miasma" an infectious matter arising outside the body. He also does not deny that possibly some of these matters may spread in both ways, and if so, the old term, "contagious-miasmatic diseases," would be rightly applied to them, but, as he finally remarks, it must be proved that diseases do actually spread in both ways, and he evidently holds it to be unlikely. Pettenkofer has thus stated again the old controversial phrases of the contagious and infectious conflicts of former days. He points out that, if there be a "contagious-miasmatic disease" (in the true sense of the word), it must be always possessed of the same properties, and must spread in both ways. We cannot suppose, he considers, that it may now spread entirely miasmatically, and at another time entirely contagiously.

He then observes that cholera, typhoid and yellow fever have many similarities in their spread. They are, he considers, importable, but not contagious diseases, but he refers for this opinion to some very weak evidence respecting yellow fever. Transportality of cholera by men and human intercourse, which is, he says, undoubted, is not to be taken as equivalent with contagious. There are places free from cholera, and which remain free; however frequently Lyons has been invaded by choleraic persons, no epidemic has ever occurred there. He argues this point, and the spread on board ship, at some length, and finally states that in the course of the observations he has made, his opinions have been altered in many ways; at first he stood, like so many others, with a predilection for the contagionist side, but was gradually, by the continued pressure of facts, driven from that position, and now believes that the customary contagious doctrine has been the greatest hindrance in our search after the nature of cholera, since our glances have not fallen on the right point, but in directions where what we seek is not to be found. He concludes "that cholera is carried by traffic, but is not on that account a contagious disease, that the causes of the increase of the cholera infection-matter, are to be sought in the surroundings of men, and not in the men themselves."

Of these surroundings he attaches, he says, an essential part to the soil. * * * * * He goes on emphatically to repeat the opinion he stated at the Cholera Conference at Weimar, in 1867: "We know that cholera is spread by traffic; we know that other additional circumstances are necessary, in order that an epidemic shall arise, and many men fall sick in one place. If only in a single case the participation of the ground is a matter which is indifferent, the same must be allowed in all other cases. I consider at present that those cases which appear as if the ground were unnecessary, are not properly analyzed. If we give up the influence of the ground in a single case, then such influence is no longer necessary for all other cases."

With regard to the "drink-water hypothesis," Pettenkofer says he has proved that in many and heavy epidemics, the participation of the drinking-water was impossible. As he was driven to seek for other local influences in these cases, so he lost the right (according to his view) to lay more weight on the water hypothesis in those cases in which it was not disproved, than in those in which the influence of the drink-water was shown to be evil. He thus found himself always thrown back on the ground, as the seat of the local condition which determined the epidemic.

In expressing himself thus, Pettenkofer appears to disregard the strong positive evidence which has been accumulated, to show that the drink-water may carry the agent. He can undertake to say, and no doubt rightly, that in some cases the drinking-water was not in fault, but he stretches his inference too far, and nothing appears to justify him in the opinion which he seems to hold so strongly, viz., that there can be only one mode of spread. Pettenkofer then passes on to the consideration of cholera on board ship, which Sander considers as fatal to the ground and ground-water theory. Pettenkofer does not allow this, and enters on a long discussion. His argument appears to be this: in no case is the spread of cholera so infrequent as on shipboard, although the conditions there are more favorable to the spread of a contagious disease than in other cases. The specific cause (*die specifische ursache*) of cholera is from time to time spread by means of human traffic from India, or from other endemic sites. In this spread there is (besides individual disposition,) "a time and place disposition." There are places where cholera, often introduced, never spreads; there are times when in a place which it is known can have epidemics, it does not prevail.

Now, in ships, the cholera is introduced from land, but in by far the greater number of cases of ships at sea, the ship is like one of those places on land which want the local disposition, and which cannot, therefore be attacked. The attacks of cholera on shipboard die out, and generally restrict themselves to persons who, in all probability, have been infected on shore. In cases in which considerable outbreaks have occurred on shipboard, the cases are usually confined to those who have been in connection with some particular place on shore; it attacks the passengers and not the crew, or one company of soldiers and not another, etc.

And yet he admits that there are some cases of real epidemic outbreaks of cholera on board ship. If we are to be consistent, and not to give up the etiological rule of the identity of the causes of cholera on land and at sea, we ought to ask, it is said, how the conditions of time and place which are necessary on shore happen in these particular cases to appear on board ship? No doubt the hypothesis of contagion would most easily explain these instances, but then by far the greater number of attacks on shipboard are not consistent, and all rational grounds are wanting to connect such epidemic outbreaks with contagion.

If cholera on board one ship has a contagious character, what prevents cholera from being contagious in all ships in which the infectious matter is introduced? * * * * There is something special on board these ships with epidemic outbreaks, which makes the part they play identical with that which the ground plays in epidemic outbreaks on shore. What that is, is quite unknown. We must renounce, with our present small knowledge of cholera, any presumption of being able to explain such occurrences now, but ships offer one of the best means of investigating certain points in the history of cholera.

If we only knew why the cholera infection material is so seldom operative on shipboard, we should be able to apply this fact at once to the case of places on land which enjoy immunity.

Passing on from this point, Pettenkofer refers to another criticism made upon his theory, viz., that he holds the opinion that the "cholera germ" (cholera keim) can increase only in the ground; he has expressly stated the contrary.

If $x$ be taken to mean the "cholera germ," which is brought by human intercourse, and $y$ the favoring local condition which allows it to grow, all he has contended for is, that this condition $y$ is somehow or other furnished by the ground; but $x$ and $y$ may meet either in or out of the ground, either in the body or out of it.

A third reproach which is made—viz., that he has not given accurate and constant signs of a cholera ground, and has not precisely stated the necessary degree of the ground-water, *i. e.*, when the ground is too dry and when too moist to allow an epidemic of cholera to occur—Pettenkofer admits to be true; he can only say, with Pio Nono, "Non possumus." We cannot precisely state these facts; we all have much to learn and much to forget; but it would be a great error, he thinks, not to discuss things because they cannot be rigidly defined.

He concludes his long and interesting article with a few remarks on the mode of investigating the part which the ground plays, and of the measures which should be adopted to check an outbreak of cholera.

If now I attempt to analyze Pettenkofer's views, I do so more with the view of attempting to define the course which inquiry should take, than with the intention of offering any decided opinion on the question of the necessary participation of the ground.

1. The point which Pettenkofer holds most decidedly, as well as Sander and Kuchenmeister, is that there is a specific cholera-producing substance which is spread only by human intercourse. Although he refers to Bryden's "Monsoon theory," and even uses the term himself, I gather that he does not mean that the cholera poison is carried by the wind, but merely that the monsoon gives favoring conditions, such as rain.

2. He then thinks that this cholera virus or miasm cannot, by itself, produce cholera in any case, but must meet with some unknown local condition or conditions in the ground or out of it, but if out of it, still furnished by the ground. If $x$ represent the unknown cholera miasm, and $y$ the unknown local conditions, then $x$ must meet $y$ to produce the agent which in a susceptible person can cause cholera. The reason which appears to have led Pettenkofer to adopt this opinion, is the fact that cholera sometimes spreads, and sometimes does not spread.

There are some places, with plenty of susceptible people, in apparently very bad sanitary condition, among whom the "cholera germ" is introduced, and yet who remain untouched; in other cases a wide spread epidemic follows. The old explanation of an epidemic constitution is untenable, as the places may be close together, the time and season may be the same, and all conditions apparently equal; yet they are not equal, for in one place, A, the disease spreads, and in the other, B, it does not. To use Pettenkofer's language, the unknown condition $y$ exists in A, but not in B. Impressed by the undoubted fact, Pettenkofer has adopted the conclusion that *y* is always *necessary*—that it is not simply a favoring condition, but that it is essential to the formation of that poison. * * * * It is not denied by the opponents of Pettenkofer that in a vast number of cases something else is operative than the mere introduction of the "cholera germ"; that some favoring conditions are in action, without which, in most cases, cholera will not widely spread; and that there may sometimes be influences from the ground; but they refuse to admit that Pettenkofer is justified in his absolute assertion of the necessity of ground influence.

3. Having thus, in his own mind, become persuaded that the "cholera germ" will not alone produce cholera, but must have local conditions in order to develop into the actual poison, Pettenkofer's third stage of argument is to show that these local conditions are connected with the ground. He is led to the ground by two roads: first, by exclusion, as he finds no other constant condition; second, by examination of the ground, which shows, he believes, that all places favorable to the spread of cholera exhibit certain characteristics of aggregation, moisture, and rise and fall of the ground, or subsoil water.

With regard to the first of these arguments, it is open to anybody to hold that Pettenkofer has too much neglected the introduction of cholera by water, food, and perhaps air; and, as respects the second argument, it cannot be doubted that at present the evidence which Pettenkofer, with such immense industry, has been able to array falls far short of demonstration; and that the decision whether the condition of the ground is really a matter of moment in the spread of cholera, and, if so, what is the exact condition which is thus favorable, are points which have still to be decided by the evidence alone admissible in scientific inquiries, viz., a sufficient number of well authenticated facts.

To me it certainly appears, at the present moment, that Pettenkofer's ground and ground-water theory is less probable than it appeared to be some years back. That the ground has an influence is highly probable, and is in fact a very old opinion; but when it is seen how very indefinite even Pettenkofer's own evidence is, and how uncertain are some of the cases he relies upon, I think it must be admitted that the enigmatical spread of cholera has not been cleared up by him. Kuchenmeister's views, on the other hand, seem more in accordance with facts.

If, without presumption, I may venture to criticise Pettenkofer's mode of looking at this subject, I should say he has allowed himself to be too much impressed by the instances in which cholera does not spread after introduction. In attempting to explain, instead of simply admitting that we cannot yet explain such cases, he has passed beyond the boundary of ascertained facts, and in so doing appears to me to have lost his hold on what are really truths.

The view which looks on the cholera-poisoning as being carried by men, and then increasing and spreading more or less in various ways, by water, air, or food, according as it meets with favoring conditions, is more in accordance with facts than the view which assumes that two unknown quantities must be brought together in order to evolve a third.

Report on Hygiene for the year 1872, by Dr. E. A. Parkes, in British Army Medical Report for 1871. London, April, 1873.

